# Case report: Directional infusion of peripheral blood stem cells into the necrotic zone in femoral heads through the medial circumflex femoral artery: A tracing study

**DOI:** 10.3389/fmed.2022.945268

**Published:** 2022-08-18

**Authors:** Qiang Mao, Weijie Shao, Shuaijie Lv, Peijian Tong, Bangjian He

**Affiliations:** ^1^Department of Orthopedics, The First Affiliated Hospital of Zhejiang Chinese Medical University, Hangzhou, China; ^2^The First School of Clinical Medicine, Zhejiang Chinese Medical University, Hangzhou, China

**Keywords:** peripheral blood stem cells, osteonecrosis of the femoral head, medial circumflex femoral artery, ^18^F-FDG, cell tracking

## Abstract

**Objective:**

This study aimed to explore whether peripheral blood stem cells (PBSCs) infused through the medial circumflex femoral artery to treat osteonecrosis of the femoral head (ONFH) could migrate into the necrotic area of femoral head.

**Methods:**

We collected PBSCs from a patient who had bilateral ONFH by apheresis technique using COBE spectra apheresis system (COBE BCT Inc, Lakewood, CO, USA) after subcutaneous injections of granulocyte-colony stimulating factor (G-CSF) at a dosage of 10 μg/kg for 4 days to mobilize PBSCs. After that, 100 MBq 2-[^18^F]-fluoro-2-deoxy-D-glucose (^18^F-FDG) was used to label PBSCs. ^18^F-FDG labeled PBSCs were infused into the left femoral head *via* the medial circumflex femoral artery to treat ONFH. Then the patient was underwent three-dimensional positron emission tomography (3D-PET) examination 60 min after cell infusion to monitor the biological distribution of ^18^F-FDG-labeled PBSCs, and to observe whether the transplanted PBSCs could migrate into the necrotic area of femoral head.

**Results:**

The total number of monouclear cells in the peripheral blood stem cell suspension was 1.95 × 10^8^ which contained 2.20 × 10^6^ CD34^+^ cells. The activity of ^18^F-FDG in the labeled cells was 1.8Bq/10^3^ monouclear cells. 3D-PET imaging showed that ^18^F-FDG radioactivity was detected in the necrotic area of femoral head, acetabulum and femoral bone marrow cavity after transplantation of ^18^F-FDG-labeled PBSCs via the medial circumflex femoral artery. It is worth noting that although PBSCs labeled with ^18^F-FDG were widely distributed around the hip, such as femoral bone marrow cavity, femoral head and acetabulum, PBSCs were generally located in the necrotic area of femoral head.

**Conclusions:**

PBSCs could enter into the femoral head and migrate into the necrotic field of femoral head participating in the repair of osteonecrosis after infusion through the medial circumflex femoral artery.

## Introduction

Osteonecrosis of the femoral head (ONFH) is a progressive pathological process characterized by bone cell death due to an interruption of the blood supply to the femoral head (1). ONFH frequently progresses to bone destruction and even femoral head collapse, which often induces unbearable hip pain and hip joint function impairment. At this point, the only therapy recommendation is to carry out a total hip arthroplasty (THA) for pain relief and reconstruction of hip joint function (2). However, artificial joints can not be expected to last a lifetime, especially for young patients, because of implant failure (3). Therefore, preserving own femoral head is the ultimate therapeutic purpose of ONFH (4). Early intervention prior to femoral head collapse is the key for the success of joint-preserving procedures (5–8).

To date, the early management approaches of ONFH remain controversial. Owing to the absence of progenitor cells, the reparative reaction around necrotic area is insufficient (9–11). Hence, the optimal treatment protocol should provide opportunity for the biologic repair in the necrotic lesion site (4). Transplantation of stem cell has been applied to enhance the biologic repair process in the osteonecrosis, and favorable results have been reported in patients with ONFH (11–14). It has been demonstrated that stem cell transplantation can alleviate pain and ameliorate the function, activity and motion of the affected hip (11–14). Granulocyte colony stimulating factor (G-CSF) is well known for its ability to mobilize bone marrow mesenchymal stem cells into peripheral circulation (4). The medial circumflex femoral artery is the main vessel which nourishes the femoral head (15). Based on our previous research, the favorable efficacy and the absence of adverse events have suggested that mobilization of bone marrow mesenchymal stem cells into peripheral circulation using G-CSF and subsequent implantation of collected peripheral blood stem cells (PBSCs) from peripheral circulation is a feasible intervention to slow and possibly reverse the effects of early and intermediate stages of ONFH (4). In previous study, we have confirmed that medial circumflex femoral artery perfusion of PBSCs can delay the progression of ONFH and medial circumflex femoral artery perfusion of PBSCs is safe (4).

The clinical efficacy of PBSCs may be closely correlated with the fate of PBSCs implanted into femoral head. Animal study show that fluorescently-labeled stem cells, injected intravenously, were found in the necrotic area of femoral head (14). In another research, to investigate the fate and distribution of stem cells, stem cells were intra-arterially infused to deal with ONFH in dog after labeled by BrdU, and the data indicated that intra-arterially perfused stem cells could migrate into the necrotic field of femoral heads and differentiate into osteoblasts, thus improving the necrosis of femoral heads (16). However, it is uncertain whether PBSCs can enter into the necrotic area of femoral head in patients with ONFH after the medial circumflex femoral artery perfusion of PBSCs. Stem cell tracing is a complex and challenging process that aims to assess homing, distribution and differentiation of stem cells. Stem cell tracing based on genetically modified cells has not been applicable to human subjects on account of the potential risk of genetic modification (17). Because of minimal toxicity, many radioisotopes such as indium, technetium and 2-[^18^F]-fluoro-2-deoxy-D-glucose (^18^F-FDG) have been clinically applied for stem cell tracing (17–19). Ultrastructural, biologic and functional properties of the stem cell are not influenced by ^18^F-FDG labeling (20). The high spatial resolution and high sensitivity of pathree-dimensional positron emission tomography (3D-PET) makes it to be an ideal imaging method for stem cell tracking (17). A report indicated that ^18^F-FDG-labeled PBSCs PET could be used to assess the tissue distribution (17). Thus, we have conducted the study that PBSCs were labeled with ^18^F-FDG and infused into femoral head *via* medial circumflex femoral artery, subsequent 3D-PET imaging was performance for patient to determine whether PBSCs can enter into the necrotic area of femoral head.

## Materials and methods

### General information

**Figure 1:**
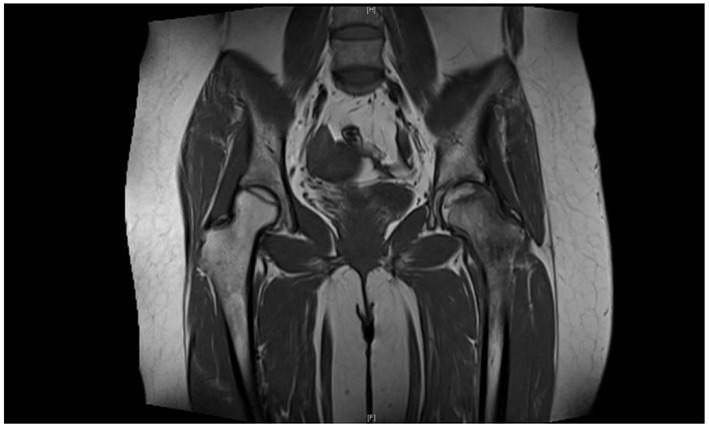
Bilateral hip MRI show bilateral ONFH.

A 60-year-old female patient developed bilateral hip joint pain of unknown origin. No attention was paid to this symptom at the time, but the pain gradually worsened. She presented to a local hospital and was diagnosed with bilateral femoral head necrosis, and received unspecified intermittent treatment. The patient had a history of rheumatoid arthritis and glucocorticoid use. She presented to our orthopedic clinic with severe pain in both hips on December 9, 2013. Hip magnetic resonance imaging (MRI) showed bilateral ONFH (Figure 1). The clinical diagnosis was bilateral ONFH(Ficat II). The patient gave informed consent for the study.

### Collection of PBSCs

**Figure 2:**
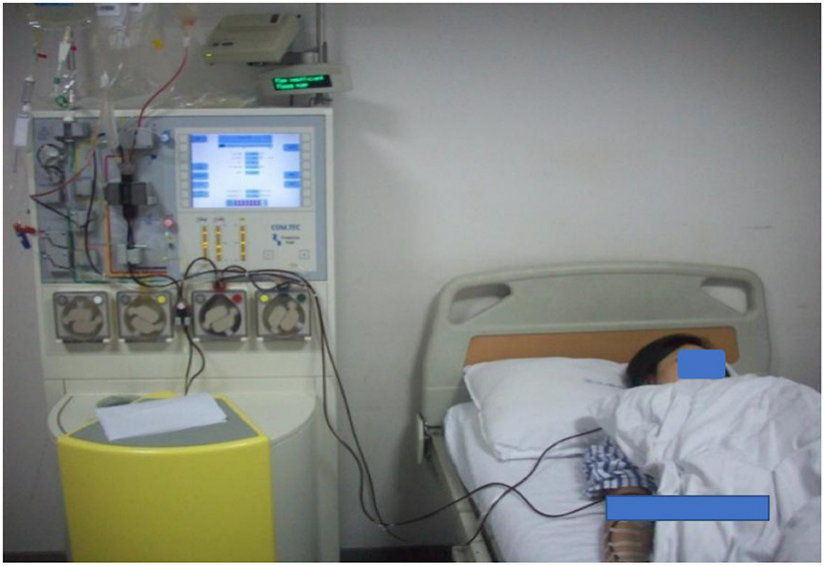
PBSCs were collected from patients. After mobilization, PBSCs were harvested from peripheral circulation of the patient by apheresis technique using COBE spectra apheresis system (COBE BCT Inc, Lakewood, CO, USA) according to the mononuclear cell collection procedure as recommended in the manual.

Before cell collection, G-CSF was subcutaneously injected at a dose of 10 μg/kg body weight, once a day for 4 days to mobilize bone marrow mesenchymal stem cells into peripheral circulation. After mobilization, PBSCs were harvested from peripheral circulation of the patient by apheresis technique using COBE spectra apheresis system (COBE BCT Inc, Lakewood, CO, USA) according to the mononuclear cell collection procedure as recommended in the manual (Figure 2) (21). The number of CD34^+^ cells in the cell suspension was calculated.

### Labeling of PBSCs

20 mL of PBSCs Suspension Were Labeled With ^18^F-FDG. PBSCs Were Incubated With 100 MBq ^18^F-FDG (Active Amount = 10 MBq/mL) in Serum-Free Phosphate-Buffered Saline (PBS) (pH 7.2) Containing 10 U/mL Heparin and 0.1 U/mL Recombinant Human Insulin Under Aseptic Conditions at 28°C for 30 Min. The Incubation System Was Shaken Gently. The Unbound ^18^F-FDG Was Removed Through three Times Centrifugation for 2 Min per Session at the Centrifugal Forces of 7, 27, and 60 g, Respectively and Cleaning Procedures Using Heparinized PBS. The ^18^F-FDG Labeled PBSCs Were Resuspended With Heparinized Saline (10 U/mL).

### Intra-arterial infusion and monitoring of PBSCs

The procedure of PBSCs transplantation has been mentioned in our previous study and briefly described as follow (22): The PBSCs were injected into femoral head *via* medial circumflex femoral artery after femoral artery puncture. Femoral artery puncture was operated with the Seldinger technique. When a guide wire was inserted and reached the heterolateral femoral artery, a 5.0 Cobra catheter was introduced into the heterolateral femoral artery along the guide wire.Then, Omnipaque was transfused into the artery with an infusion speed of 5 ml/s for angiography to discover the medial circumflex femoral artery. Finally, the labeled PBSCs were perfused into the left femoral head through the medial circumflex femoral artery. The position of the Cobra catheter was ensured with fluoroscopy. Then, 3D-PET imaging was performed (60 min after PBSCs transplantation) to observe the PBSCs distribution.

## Results

### Collection and labeling of PBSCs

The total number of monouclear cells in the peripheral blood stem cell suspension was 1.95 × 10^8^ which contained 2.20 × 10^6^ CD34^+^ cells. The activity of ^18^F-FDG in the labeled cells was 1.8Bq/10^3^ monouclear cells.

### Distribution of PBSCs labeled with ^18^F-FDG

**Figure 3:**
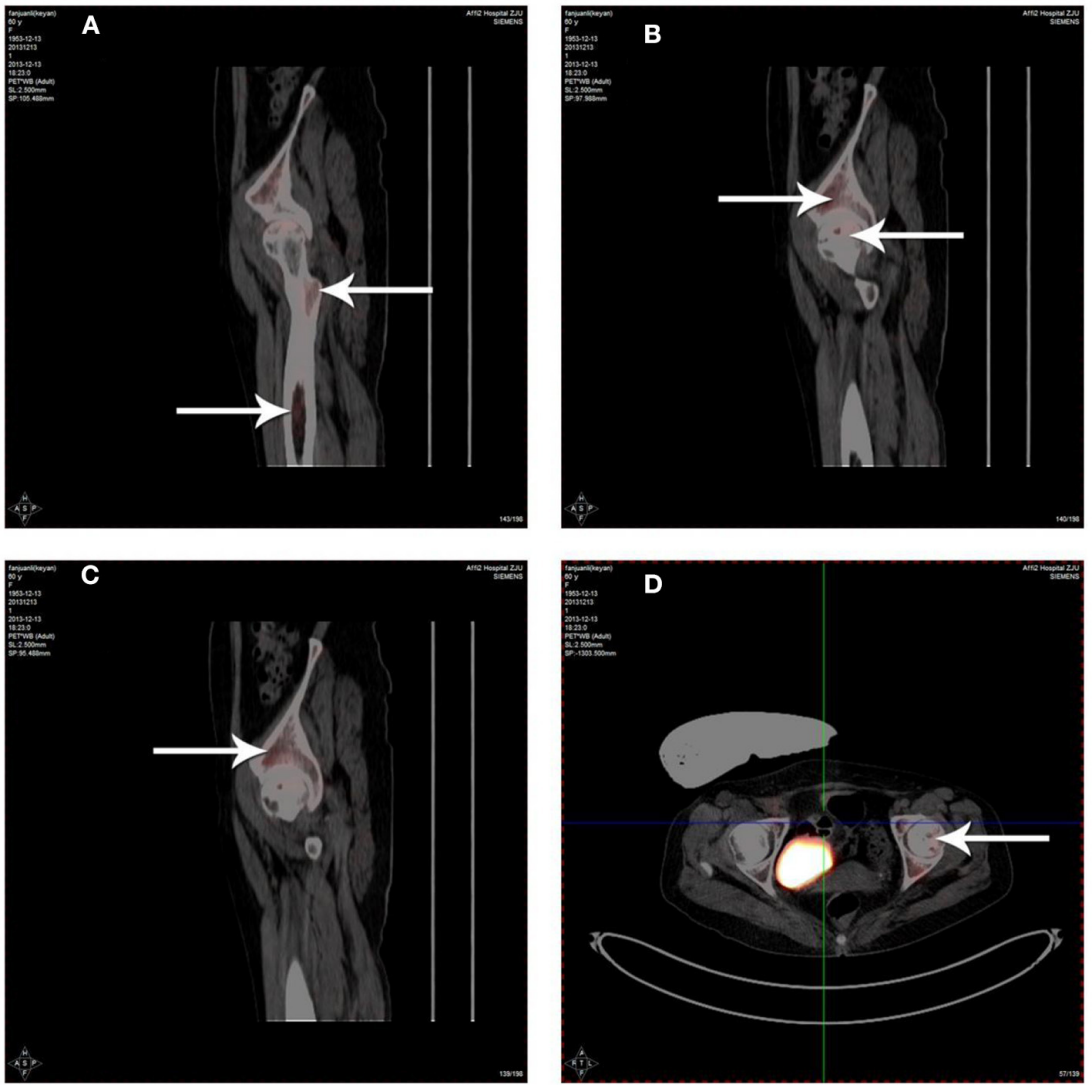
Distribution of ^18^F-FDG labeled PBSCs after infusion via the medial circumflex femoral artery. The ^18^F-FDG labeled PBSCs could be observed in the femoral bone marrow cavity **(A)**, necrotic area of the femoral head **(B,D)** and acetabulum **(C)**. The ^18^F-FDG labeled PBSCs could be seen in necrotic area of the femoral head indicating that PBSCs could enter into the femoral head and migrate into necrotic field of the femoral head participating in the repair of osteonecrosis after infusion through the medial circumflex femoral artery.

Radioactivity of ^18^F-FDG was detected in the femoral bone marrow cavity (Figure 3A), necrotic area of the femoral head (Figures 3B,D) and acetabulum (Figure 3C) by 3D-PET imaging after PBSCs transplantation via the medial circumflex femoral artery. The data showed that although PBSCs labeled with ^18^F-FDG were widely distributed around the hip, such as femoral bone marrow cavity, femoral head and acetabulum, PBSCs were generally located in necrotic area of the femoral head. These revealed that PBSCs could enter into the femoral head through the medial circumflex femoral artery and migrate into the necrotic field of the femoral head after intra-arterial infusion.

## Discussion

Stem cell transplantation is a promising treatment strategy for ONFH (5, 23). Numerous animal and clinical studies have demonstrated that stem cell transplantation can indeed improve hip function and delay the progression of ONFH (5, 11, 22, 24–28). However, studies on stem cell labeling and tracer techniques for evaluating stem cell implantation, distribution, survival, migration, and differentiation lag behind and have become a bottleneck in mechanistic studies of stem cell therapy (29). To clarify the mechanism of stem cell transplantation therapy and monitor the survival of stem cells in target tissues, non-invasive *in vivo* tracing of transplanted stem cells is required. Non-invasive imaging technology is crucial for clinical applications of stem cell research (30). Cellular imaging can trace the migration, distribution, and survival of cells *in vivo*, and evaluate their therapeutic effects (31).

Current cell and molecular imaging techniques for stem cell tracing include MRI, nuclear medical imaging, optical imaging, ultrasonic and photoacoustic imaging, magnetic particle imaging, and multimodal imaging. Optical imaging and magnetic particle imaging are difficult to apply clinically, while nuclear imaging and MRI have been widely used in human clinical trials. Nuclear medicine has the advantage of greater tissue penetration depth compared to MRI (32). Nuclear medicine imaging modalities include PET and single-photon emission computed tomography (SPECT). This technology uses radionuclides to label stem cells and monitors them through imaging. Quantitative analyses of cell vitality and viability can be performed with high sensitivity (33). PET (−1 cm/s) is the most sensitive cell and molecular imaging modality, with better temporal and spatial resolution than SPECT (1.0–2.0 cm/min) (34, 35). Although contemporary nanoparticle tracers have more stable and precise imaging performance, their safety needs to be improved. ^18^F-FDG, known as the “molecule of the century”, is still the most widely used radionuclide for PET (36). Both PET and ^18^F-FDG have been used to trace and evaluate the efficacy of stem cell transplantation in the treatment of heart diseases (37). They are of great value for diagnosis, prognosis, and treatment response monitoring. Therefore, in this study, PBSCs were labeled with ^18^F-FDG, and the distribution of transplanted PBSCs in the femoral head was monitored using PET.

Further analysis of radiation dose showed that, among the ^18^F-FDG labeled cells, there were 1.8 Bq/10^3^ monouclear cells, which is considered to reflect high labeling efficiency. Whether or not PBSCs perfused through the medial circumflex femoral artery can enter into the necrotic field of the femoral head and exert effects therein remains questionable. Our results showed that PBSCs could be detected in necrotic areas of the femoral head, acetabulum and femoral bone marrow cavity. Although these results suggest that the transplanted PBSCs are not exclusively concentrated in the necrotic area, i.e., are also distributed in other parts of the hip joint, it has been demonstrated that PBSCs transplanted *via* the medial circumflex femoral artery can enter and function in the necrotic area.

The main limitation of the present study was that the viability and survival time of the transplanted PBSCs were not quantified. Due to the nuclide half-life of ^18^F-FDG used (about 110 min), the limited tracer time became the main obstacle to long-term tracing, and only immediate imaging analysis of transplanted cells could be performed (26). Another disadvantage was that only a single case study was carried out. Nuclear medical imaging has disadvantages in terms of cell quantification and technical resolution. Stem cell perfusion bias may also exist in transvascularized stem cell grafts. Therefore, further studies with larger samples and more advanced tracer techniques are needed. As an example, the latest dual-mode imaging modality, comprising PET and MRI, provides anatomical information with high soft tissue contrast, as well as highly sensitive imaging and functional labeling of labeled transplanted cells in tissues.

## Conclusion

The present study used ^18^F-FDG to label PBSCs, and PET imaging to monitor perfusion through the medial circumflex femoral artery *in vivo*. The results confirmed that PBSCs transplanted through this approach can enter into necrotic areas of the femoral head. The present study suggests that PBSCs could enter into the femoral head and migrate into necrotic field of the femoral head after infusion through the medial circumflex femoral artery.

## Data availability statement

The original contributions presented in the study are included in the article/supplementary material, further inquiries can be directed to the corresponding author.

## Ethics statement

The studies involving human participants were reviewed and approved by the First Affiliated Hospital of Zhejiang Traditional Chinese Medicine University. The patients/participants provided their written informed consent to participate in this study.

## Author contributions

BH and QM conceived and coordinated the study and wrote the paper. WS, PT, and BH carried out the data collection and analysis. WS, PT, BH, and SL revised the paper. All authors reviewed the results and approved the final version of the manuscript.

## Funding

This study was supported by National Natural Science Foundation of China (No. 82074469) and Zhejiang Provincial Natural Science Foundation of China (No. LY21H270008).

## Conflict of interest

The authors declare that the research was conducted in the absence of any commercial or financial relationships that could be construed as a potential conflict of interest.

## Publisher's note

All claims expressed in this article are solely those of the authors and do not necessarily represent those of their affiliated organizations, or those of the publisher, the editors and the reviewers. Any product that may be evaluated in this article, or claim that may be made by its manufacturer, is not guaranteed or endorsed by the publisher.
